# Issue when expressing a recombinant protein under the control of p*35S* in *Nicotiana tabacum* BY-2 cells

**DOI:** 10.3389/fpls.2023.1266775

**Published:** 2023-11-13

**Authors:** Catherine Navarre, Rik Orval, Marie Peeters, Nicolas Bailly, François Chaumont

**Affiliations:** Louvain Institute of Biomolecular Science and Technology (LIBST), UCLouvain, Louvain-la-Neuve, Belgium

**Keywords:** Agrobacterium, bacterial transposon, LBA4404, Ti plasmid, BY-2 cells

## Abstract

Several recombinant proteins have been successfully produced in plants. This usually requires *Agrobacterium*-mediated cell transformation to deliver the T-DNA into the nucleus of plant cells. However, some genetic instability may threaten the integrity of the expression cassette during its delivery via *A. tumefaciens*, especially when the protein of interest is toxic to the bacteria. In particular, we found that a Tn3 transposon can be transferred from the pAL4404 Ti plasmid of *A. tumefaciens* LBA4404 into the expression cassette when using the widely adopted *35S* promoter, thereby damaging T-DNA and preventing correct expression of the gene of interest in *Nicotiana tabacum* BY-2 suspension cells.

## 1 Introduction

The production of recombinant proteins using transient and stable expression in plants has become an alternative to more conventional animal cell line platforms (Schillberg and Spiegel, 2022). Also, expression in plants offers the possibility for metabolic engineering of plant bioactive pathways (Reed and Osbourn, 2018). *Agrobacterium tumefaciens*-mediated transformation is the method of choice to deliver the genetic material into plants. The gene(s) of interest under the control of suitable promoter and terminator sequences is inserted between the left and right borders delineating the T-DNA in a binary vector. Generally, the *35S* promoter from Cauliflower mosaic virus (CaMV), and its derivatives like the enhanced CaMV *35S* promoter consisting of a 250 bp tandem duplication of sequence upstream of the core promoter, is preferred to direct constitutive expression of transgenes in both stable and transient expression systems (Amack and Antunes, 2020). The *35S* promoter is active in a large number of plant species, both dicots and monocots. However, it is important to bear in mind that this promoter is also active in *Escherichia coli* (Assaad and Signer, 1990) and *A. tumefaciens* (Ohta et al., 1990; Vancanneyt et al., 1990), which can be of concern when the gene to be expressed is toxic to the bacteria. Indeed, in this case, the binary plasmid is generally mutated or rearranged to prevent the expression of the toxic protein. The toxic effect on the host bacteria can be reduced by lowering the culture temperature. In addition, plant introns have been previously proposed to stabilize plasmids in bacteria (Johansen, 1996).

Here, we examined the fate of two expression cassettes corresponding to a viral envelope glycoprotein (gP) when transferred into *A. tumefaciens* and delivered into BY-2 cells. We found that a replicative DNA transposon was integrated within *Agrobacterium* LBA4404 into the T-DNA when the *gP* coding sequence was under the control of *p35S*, but not if a plant-specific promoter was preferred.

## 2 Method

### 2.1 Construction of the genes and vectors

To design the binary vectors, the *gP* expression cassettes were constructed using the Golden Gate Modular Cloning (MoClo) assembly method (Weber et al., 2011) and DNA parts from the MoClo Plant toolbox (Engler et al., 2014). Oligonucleotides encoding the *Medicago sativa* protein disulfide isomerase (PDI) signal peptide flanked by BpiI recognition and restriction sites (PDIforward/PDIreverse; **Table S1**) were hybridized and cloned into the *pICH41258* level 0 acceptor plasmid. Oligonucleotides encoding a 6xHis tag flanked by BpiI recognition and restriction sites (Histagforward/Histagreverse; **Table S1**) were hybridized and cloned into the *pAGM1301* level 0 acceptor plasmid. The promoter of plasma membrane *ATPase 4* gene from *Nicotiana plumbaginifolia*, reinforced with two *CaMV35S* enhancers (De Muynck et al., 2009), contained four BpiI recognition sites, which had to be removed to avoid hampering the efficacy of subsequent Golden Gate reactions. Appropriate mutations were therefore introduced by PCR using three primer sets (PMA1/PMA2, PMA3/PMA4, PMA5/6; **Table S1**). The three fragments were flanked by BpiI recognition and restriction sites, enabling them to be reassembled in the *pICH41295* level 0 acceptor plasmid. The sequence of the gP ectodomain (Ala21-Gly419) optimized for plant expression was first amplified by PCR with the primer set gPforwardnoSP/gPreverse (**Table S1**) and cloned into *pGEMT-Easy* vector. The sequence of the glycoprotein cannot currently be disclosed for intellectual property reasons. The sequence of the *Saccharomyces cerevisiae PMP1* terminator was amplified by PCR as a 0.6 kb fragment using the primer set tPMP1forward/tPMP1reverse (**Table S1**) and a pEMBL12- vector containing the *PMP1* gene (Navarre et al., 1994), and cloned into the *pGEMT-Easy* vector. The *pGEMT-Easy* vectors were sequenced and further used as level 0 Golden Gate modules.

By a Golden Gate reaction, the plasmids containing the enhanced *35S* promoter (a tandem duplication of 327 bp sequence upstream of the core promoter (-90 region) + 5’ untranslated Ω leader (*pICH51288*), the sequences encoding the PDI signal peptide, gP and the 6xHis-tag, and the *PMP1* terminator were digested with BsaI and then assembled into the level 1 acceptor position 2 Golden Gate *pICH47742*. Alternatively, the plasmids containing the *PMA4* promoter, the sequences encoding the PDI signal peptide, gP and the 6xHis-tag, and the *PMP1* terminator were digested with BsaI and then assembled into the level 1 acceptor position 2 Golden Gate *pICH47742*.

*N. tabacum RB7* SAR genetic insulator was PCR amplified from the pPZP-nptII-mcherry-gB-gB-SAR binary vector (Herman et al., 2023) using primers flanked by BpiI recognition and restriction sites (SARforward/SARreverse; **Table S1**). The PCR fragment was then cloned into *pICH41331*. By Golden Gate reaction, the SAR sequence was digested with BsaI and ligated into the level 1 acceptor plasmid level 3 *pICH47751*. The *hptI* hygromycin resistance gene under the control of the *NOS* promoter and terminator was PCR amplified with primers flanked by BpiI recognition and restriction sites (hptIforward/hptIreverse; **Table S1**) from the pPZP-hptI-HIgG1-LoBM2 binary vector (Magy et al., 2014) and then cloned into the level 1 position 1 vector *pICH47732*. A *nptII* kanamycin resistance cassette under the control of the *NOS* promoter and the *OCS* terminator (*pICSL70004*) was transferred into the level 1 acceptor position 1 *pICH47732* by Golden Gate with BsaI.

**Figure 1:**
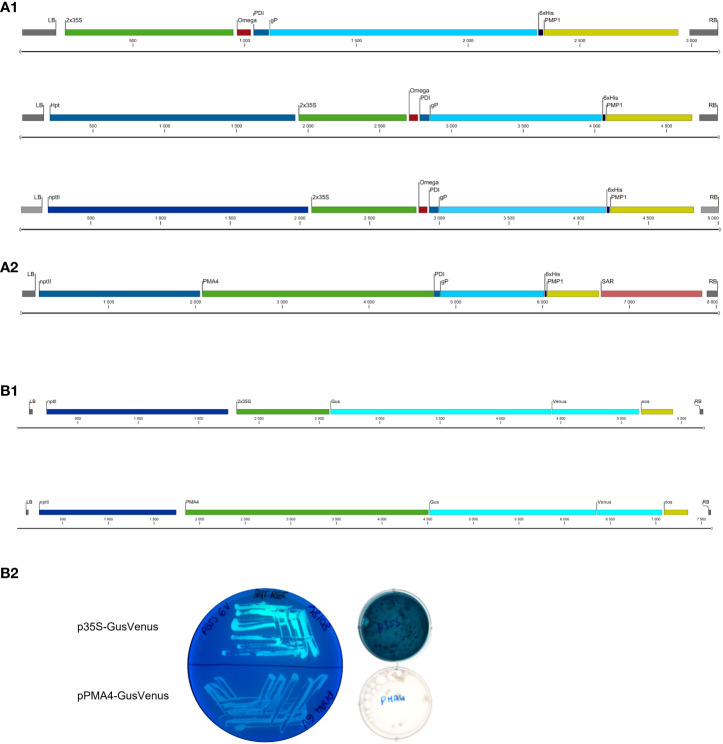
Schematic representation of the expression cassettes within the T-DNA in the binary plasmids used. **(A)** gP expression cassettes **(A1)** p35S-SPPDI-gP-6His-tPMP1. **(A2)** pPMA4-PDI-gP-6His-tPMP1 **(B)** GusVenus expression cassettes **(B1)** p35S-GusVenus-tnos; pPMA4-GusVenus-tnos **(B2)** Venus fluorescence and Gus staining in *Agrobacterium* cells.

The two gP expression cassettes (*p35S-PDI-gP* and *PMA4-PDI-gP*) were moved by Golden Gate reaction to a level M multigene vector containing a spectinomycin resistance gene by digestion with BpiI followed by ligation in the acceptor vector *pAGM831* (*PMA4-PDI-gP*) or *pAGM8043* (*p35S-PDI-gP*) (**Figure 1**). For the selection of stable BY-2 transformants, the cassette containing the hygromycin phosphotransferase (*hpt*) gene or the neomycin phosphotransferase (*nptII*) gene was added in position 1. For the *PMA4-PDI-gP* construct, the SAR cassette was also added in position 3. Finally, to close the level M acceptors, the level M end-link 2 (*pICH50881*) was used for *p35S-PDI-gP* construct whereas the level M end-link 3 (*pICH50892*) was used for *PMA4-PDI-gP* construct.

The binary plasmids pPZP-nptII-pPMA4-GusVenus and pPZP-nptII-p35S-GusVenus in which the GusPlusVenus bi-functional reporter construct was either under the control of the enhanced *PMA4* promoter of *N. plumbaginifolia* or the enhanced *35S* promoter (a tandem duplication of 327 bp sequence upstream of the core promoter (-90 region) + the *35S* core promoter (-90,+1)) were already available in our lab (**Figure 1B1**) (Navarre et al., 2011).

### 2.2 Transformation of Agrobacterium tumefaciens

The binary vectors corresponding to gP expression cassettes were transferred into the electrocompetent *A. tumefaciens LBA4404 virG* strain (van der Fits et al., 2000) or GV3101::pMP90, and selected on 2YT medium (16 g/L Bacto-tryptone, 10 g/L Bacto Yeast Extract, 5 g/L NaCl, 2 g/L Glucose, 0.4 g/L MgSO4) containing rifampicin, gentamicin and spectinomycin (50 µg/mL). Extraction and purification of plasmids from *A. tumefaciens* were carried out using the SMARTPURE SK-PLPU-100 kit (Kaneka Eurogentec, Seraing, Belgium). The binary plasmids purified from *A. tumefaciens* transformants were re-transformed into *E. coli* TOP10 cells, selected for spectinomycin resistance and sequenced.

Both vectors containing to GusVenus cassette were transferred into the electrocompetent *A. tumefaciens* LBA4404 virG strain. Detection of Venus expression was visualized with an Amersham Imager 600 using a light source and the Cy2 (480 nm) and CY3 (520 nm) filters. For Gus *in vivo* staining, late logarithmic *Agrobacterium* cultures were stained for 1 h with 2 mM of the indigogenic substrate X-Gluc (5-bromo-4-chloro-3-indolyl beta-D-glucuronic acid cyclohexylammonium) as reported by (Vancanneyt et al., 1990).

### 2.3 Transformation of BY-2 cells

The *XylT/FucT* KO BY-2 cell line, knocked out for genes encoding β(1,2)-xylosyltransferases and α(1,3)fucosyltransferases (Mercx et al., 2017), was used to generate transgenic cell lines expressing gP via *A. tumefaciens* LBA4404VirG-mediated stable transformation of BY-2 cells as described (Navarre and Chaumont, 2022). Biolistic particle delivery in BY-2 cells was carried out with a Biolistic PDS1000/He device (Bio-Rad, Hercules, CA, USA) as previously described (Herman et al., 2021).

### 2.4 Western blotting analysis of proteins

Samples of total soluble proteins (TSP) were analyzed by SDS-PAGE (pre-casted 4–20% polyacrylamide, Kaneka Eurogentec, Seraing, Belgium) after denaturation for 5 min at 100°C in Laemmli buffer containing 0.1 M DTT, and transferred onto a PVDF membrane (Bio-Rad, Trans-Blot Turbo, 1704156, Hercules, CA, USA). The PVDF membrane was incubated with a rabbit polyclonal antibody against gP (proprietary antibodies, 1/1,000) followed by anti-rabbit, alkaline phosphatase (AP)-coupled polyclonal antibodies (Sigma-Aldrich, A3687, St Louis, MO, USA, 1/10,000). Western blots were revealed with BM Purple AP substrate, precipitating (Roche, 11442074001, Basel, Switzerland).

## 3 Results

**Figure 2:**
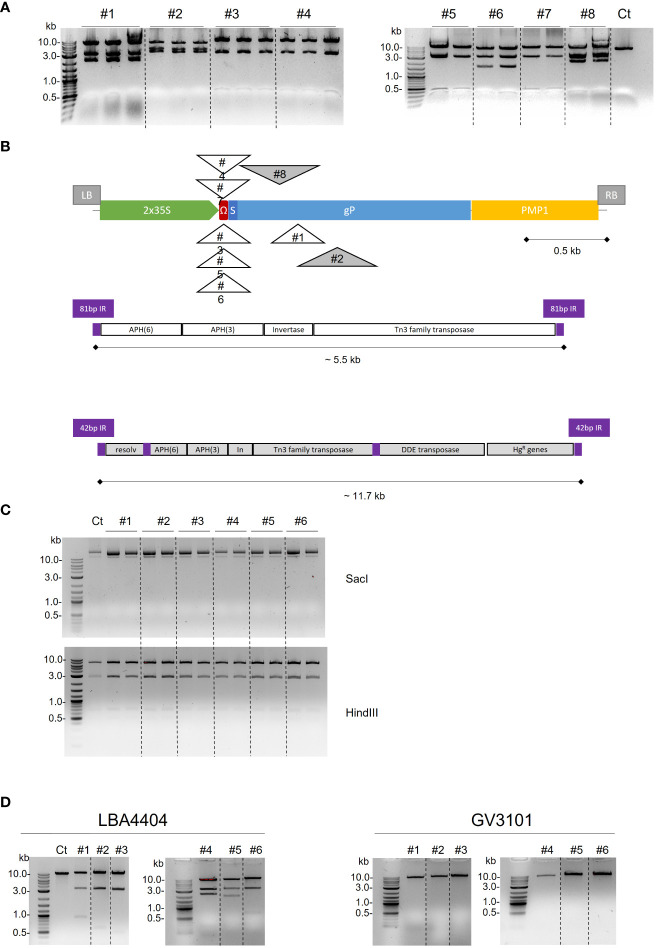
Alteration of binary plasmid p35S-gP T-DNA after *A. tumefaciens* passage. **(A)** Electrophoretic analysis of the p35S-SPPDI-gP-6His-tPMP1 plasmid purified from eight *A. tumefaciens* LBA4404 transformants, retransformed in *E. coli* and digested with SacI. A control plasmid without any passage in *Agrobacterium* is used as a control (Ct). **(B)** Schematic diagram of transposon elements insertions in T-DNA of plasmid p35S-SPPDI-gP-6His-tPMP1 after passage in *A. tumefaciens* LBA4404. Both orientations of Tn3 are represented above or below the T-DNA. Genes within the 5.5 kb and 11.7 kb transposon elements are detailed. **(C)** Electrophoretic analysis of the nptII-pPMA4-SPPDI-gP-6His-tPMP1 plasmid purified from six *A. tumefaciens* LBA4404 transformants, retransformed in *E. coli* and digested with SacI or HindIII. A control plasmid without any passage in *Agrobacterium* is used as a control (Ct). **(D)** Electrophoretic analysis of the nptII-p35S-SPPDI-gP-6His-tPMP1 plasmid purified from six *A. tumefaciens* LBA4404 or six *A. tumefaciens* GV3101 transformants, retransformed in *E. coli* and digested with SacI. A control plasmid without any passage in *Agrobacterium* is used as a control (Ct).

The *gP* expression cassette was constructed by assembling the sequences encoding the signal peptide of the *M. sativa* protein disulfide isomerase (PDI), the gP ectodomain, and the C-terminal 6-His tag, under the control of the 2x*35S* promoter fused to the 5’ untranslated Ω-leader of the CaMV, and the terminator of the yeast *PMP1* gene (**Figure 1A1**). While the binary plasmid containing this *p35S-PDI-gP* construct was correctly obtained in *E. coli*, *A. tumefaciens* LBA4404 transformation led to unexpected digestion profiles that varied according to the transformant. This likely indicated that the binary plasmid containing the *gP* cassette was altered in *A. tumefaciens* LBA4404. The binary plasmids purified from eight different *A. tumefaciens* LBA4404 transformants (coming from two independent electrocompetent *Agrobacterium* batches) were retransformed in *E. coli*, analyzed by restriction analysis (**Figure 2A**), and sequenced. Sequencing with LB and internal primers showed that the insertion of an additional DNA fragment occurred within the *gP* cassette (**Figure 2B**).

For six out of the eight sequences, the left and right ends of the inserted DNA matched with two regions comprised between positions 1,066 and 6,534 of the 140-kb-long pAL4404 tumor inducing plasmid (pTi) of the *A. tumefaciens* LBA4404 strain (accession number KY000037.1) (**Figure 2B**). Analysis of the published pAL4404 annotations showed that this 5.5-kb long sequence is bordered by 81 bp inverted repeats and encodes two aminoglycoside 3’-O-phosphotransferases (APH(6)-Id and APH(3’)-Ib), a transposon DNA invertase and a transposase of the Tn3 transposon family (**Figure 2B**). This sequence corresponds to the *Tn904* transposon. In five cases out of six, the *Tn904* transposon was inserted within the 5’ Ω-leader and, in the sixth case, the insertion occurred within the *gP* sequence (Thr171).

The last two sequences showed different borders that also matched with two regions comprised between positions 1 and 11,727 of pAL4404 (**Figure 2B**). This 11.7 kb sequence is flanked by 42 bp inverted repeats and contains the same 5.5 kb sequence that was detected in the other six sequences and identified as the *Tn904* transposon, as well as mercury resistance genes and additional transposases. Both insertions occurred within the *gP* sequence (Val159 or Ile211).

Altogether, our findings indicated that the binary plasmid containing the cassette encoding the gP ectodomain under the control of the *35S* promoter was altered in *A. tumefaciens* LBA4404 possibly to prevent its expression. To confirm the activity of *35S* promoter in *A. tumefaciens* LBA4404, overnight cultures of the strain containing the gene encoding the GusVenus bi-functional reporter under the control of the 2x*35S* promoter were incubated with X-gluc, a substrate for Gus, or checked for Venus fluorescence (**Figure 1B2**). As a control, *A. tumefaciens* containing the GusVenus bi-functional reporter gene under the control of the strong constitutive *PM4* promoter from *N. plumbaginifolia* showed no activity in the same conditions.

**Figure 3:**
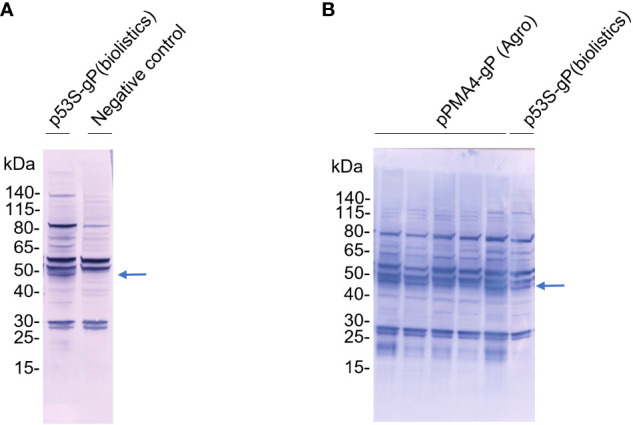
Expression of gP in XylT/FucT KO BY-2 cell line. TSP from gP transgenic cell lines obtained after biolistics or *Agrobacterium* transformation were analysed by Western Blotting using antibodies raised against gP and rabbit AP-coupled secondary antibodies. **(A)** Sixty µg of TSP from the p35S-gP transgenic cell line (Biolistics) and a transgenic control cell line expressing gB from HCMV (negative control); **(B)** Thirty µg of TSP from five independent PMA4-gP transgenic cell lines (*Agrobacterium*) and the p35S-gP transgenic cell line (Biolistics).

We therefore used biolistics to obtain stable BY-2 transformed cells without going through an *Agrobacterium* co-cultivation step. The binary plasmid containing the *PDI-gP* expression cassette as well as a hygromycin resistance marker (**Figure 1A**) was coated on golden particles and delivered into BY-2 cells by high-speed particle bombardment. A transgenic cell line selected on hygromycin-supplemented growth medium was analyzed by Western blotting using polyclonal anti-gP antibodies. Compared to the control BY-2 cell line producing the gB ectodomain from human cytomegalovirus (Herman et al., 2023), an additional band at 50 kDa was detected, corresponding to the predicted size of gP (**Figure 3A**).

An alternative to overcome the transposon issue in *A. tumefaciens* LBA4404 consisted of replacing the *35S* promoter with a plant promoter that has no activity in *A. tumefaciens*, like the plant plasma membrane proton pump ATPase *PMA4* promoter (**Figure 1A2**). The corresponding binary plasmid containing the *PDI-gP* open reading frame under the control of the *PMA4* promoter, and the kanamycin resistance marker was transferred in *A. tumefaciens*. The binary plasmids purified from six different *A. tumefaciens* LBA4404 transformants were retransformed in *E. coli*. The integrity of the binary plasmid was checked by restriction analysis (**Figure 2C**). Transgenic BY-2 cell lines were then generated via *A. tumefaciens* co-cultivation and selected on kanamycin-supplemented growth medium. A specific band at 50 kDa was detected by Western blotting analysis using polyclonal anti-gP antibodies (**Figure 3B**).

## 4 Discussion

For BY-2 cells transformation, we routinely use the *A. tumefaciens* strain LBA4404 virG (van der Fits et al., 2000), which has proved to be much more effective than others in obtaining transgenic BY-2 calli (Geelen and Inze, 2001; De Saeger et al., 2021). The LBA4404 strain directly derives from the LBA4213 strain, itself a derivative of the wild type Ach5 strain. To label the *A. tumefaciens* pTiAch5 plasmid regions, the *Tn904* transposon, coding for streptomycin resistance and originating from *Pseudomonas aeruginosa*, was inserted into the Ti plasmid pAL102 present in *A. tumefaciens* Ach5 (Klapwijk et al., 1980; Ooms et al., 1980). Among the mutants that were not affected in virulence, the strain LBA4213 harboring the pAL231 pTi mutant was used to generate the strain LB4404, which contains a large deletion in the T-DNA in its non-oncogenic pAL4404 pTi (Ooms et al., 1982). Both pAL231 and pAL4404 pTi plasmids contain the same *Tn904* insertion. Our results clearly showed that the gP-encoding cassette in the binary plasmid with a pRK2 origin was altered in *A. tumefaciens* LBA4404 by the insertion of transposons from the Tn3 family, which originated from pAL4404. This phenomenon was previously reported for a pGA472 derived binary vector, in which the Tn5393 transposon was inserted in the terminator sequence of the hygromycin-resistant selectable marker (Kim and An, 2012). Interestingly, this corruption of the expression cassette was not detected when gP was under the control of the plant *PMA4* promoter, which has no activity in *Agrobacterium*. Moreover, in all tested transformants, the *Tn904* transposon was inserted into the 5’ Ω-leader or in the *gP* open reading frame. We hypothesized that gP expression under the control of the *35S* promoter was toxic to *A. tumefaciens* LBA4404 and that a counter-selection pressure occurred in *Agrobacterium* transformants, leading to the survival of only those colonies that had undergone a transposition event leading to the inhibition of gP expression.

Actually, certain Agrobacterium strains like LBA4404 appear to be more prone to plasmid instability than others (De Saeger et al., 2021). Another strain frequently used in plant biotechnology is the strain C58-derived GV3101. We transformed the binary plasmid that contains the gP open reading frame under the control of the *35S* promoter and the kanamycin resistance cassette (**Figure 1A**) in the strains GV3101 or LBA4404, and showed no alteration of the binary plasmid restriction profile after GV3101 passage, while the plasmid was corrupted after passage in LBA4404 (**Figure 2D**). Whole sequencing of the gP expression cassette in the six binary plasmids derived from GV3101 confirmed its full integrity. These data indicate that the plasmid instability is dependent on the Agrobacterium strain and the presence of the transposon Tn904.

Given that previous data also reported that the transposon Tn5393 can be inserted into the plant genome along with the T-DNA, possibly via horizontal gene transfer (Zhao et al., 2009; Kim and An, 2012; Philips et al., 2017), caution should be taken when using *A. tumefaciens* LBA4404 T-DNA delivery.

In conclusion, this study highlights the necessity for a careful examination of each step while expressing recombinant proteins in plant cells, especially when using *Agrobacterium*-mediated transformation. More precisely, we recommend a close selection of the *A. tumefaciens* transformants when using LBA4404.The use of a promoter that shows no activity in bacterial cells should be preferred. The integration of an intron into the transgene coding sequence is another alternative. Finally, a modified *Agrobacterium* LBA4404 strain lacking the Tn904 transposon would be very useful, like in the patent recently published (Annaluru and Bass, 2022).

## Data availability statement

The data analyzed in this study is subject to the following licenses/restrictions: This article utilises proprietary data. Requests to access these datasets should be directed to Catherine Navarre, catherine.navarre@uclouvain.be.

## Author contributions

CN: Conceptualization, Data curation, Funding acquisition, Investigation, Methodology, Supervision, Writing – original draft, Writing – review & editing. RO: Investigation, Writing – original draft. NB: Investigation, Writing – review & editing. MP: Investigation, Writing – review & editing. FC: Conceptualization, Funding acquisition, Supervision, Writing – review & editing.
