# Supplementary material for: Issue when expressing a recombinant protein under the control of p35S in Nicotiana tabacum BY-2 cells
Source: Front Plant Sci. 2023 Nov 13;14:1266775. doi: 10.3389/fpls.2023.1266775 (PMC10679441; doi:10.3389/fpls.2023.1266775)
Supplement: Supplementary file 1 [file DataSheet_1.pdf]

Table S1

The primers used in this study

Primer name	Primer sequence (5'→3')
gPforward	TTGGTCTCAAGGTGCAGCTCCTAGAACTTCTTG
gPreverse	TTGGTCTCTCGAACCTCCAAGTCTAAGAGGTCCTC
tPMP1forward	GGTCTCGGCTTAAAGGGCGGAGCGAGCCATTTTATT
tPMP1reverse	GGTCTCGAGCGAGAAAAAGTAGATCATAAC
PDIforward	[Phos]AATGGCGAAAAACGTTGCGATTTTCGGTTTATTGTTTTCTCTTCTTCTGTT GGTTCCTTCTCAGATCTTCGCA
PDIreverse	[Phos]ACCTGCGAAGATCTGAGAAGGAACCAACAGAAGAAGAGAAAACAATAA ACCGAAAATCGCAACGTTTTTCGC
Histagforward	[Phos]TTCGCATCATCATCATCATCATTAA
Histagreverse	[Phos]AAGCTTAATGATGATGATGATGATG
PMA1	TTGAAGACAAGGAGGGATCGATCCTCGATCTACA
PMA2	AAGAAGACTTTGTAGACGTGGTTGGAACCTCTTCTTTTTCCACGATGCTCCTCG
PMA3	TTGAAGACAAGAGGTTCCAACCACGTCTACAAAGCAAGTGGATTGATGTGA
PMA4	AAGAAGACTTCATTACCGGATCTTCTCTGCTCC
PMA5	TTGAAGACAATACAAAGCAAGTGGATTGATGTGA
PMA6	AAGAAGACTTCCTCTTCTTTTTCCACGATGCTCC
gP200reverse	CAACCCAAGATGGTCTAAGTG
gP590forward	CAACACCTCCAAGAAGG
gP900reverse	ACAGCAAAGGAGCATCAG
SARforward	GGTCTCAGGAGTCGATTAAAAATCCCAATTATA
SARreverse	GGTCTCTAGCGACTATTTTCAGAAGAAGTTCCC
hptIforward	TTGGTCTCAGGAGCCAGGCGGGAAACGACAATC
hptIreverse	AAGGTCTCTAGCGCCCTGTCGAGGGGGGATC
